# Notes from the Field: Seroprevalence Estimates of SARS-CoV-2 Infection in Convenience Sample — Oregon, May 11–June 15, 2020

**DOI:** 10.15585/mmwr.mm6932a4

**Published:** 2020-08-14

**Authors:** Melissa Sutton, Paul Cieslak, Meghan Linder

**Affiliations:** 1 Public Health Division, Oregon Health Authority.

The first known case of coronavirus disease 2019 (COVID-19) in Oregon was diagnosed on February 28, 2020. Through May 31, a total of 4,243 COVID-19 cases in Oregon were confirmed by nucleic acid testing for SARS-CoV-2, the virus that causes COVID-19, yielding a cumulative COVID-19 incidence of approximately 0.1%.* Because this rate does not account for persons who were infected but did not seek testing (e.g., those with asymptomatic or mildly symptomatic infections), persons who chose not to be tested, or persons unable to access testing, the rate is believed to be lower than the true cumulative COVID-19 incidence in the state. A population-based seroprevalence survey can provide estimates of the cumulative incidence of infection more accurately than does nucleic acid testing by identifying additional persons who have had previous infections with SARS-CoV-2 but were not reported as COVID-19 cases. Seroprevalence estimates from several states and geographic areas within the United States vary from 1.0% to 6.9^%^ (*1*–*4*). No seroprevalence estimates for SARS-CoV-2 infection are yet available for Oregon.

To estimate the seroprevalence of infection with SARS-CoV-2 in Oregon, a cross-sectional, population-based convenience sample for SARS-CoV-2 immunoglobulin G (IgG) antibody testing was collected from mid-May through mid-June, in alignment with the World Health Organization seroepidemiologic investigation protocol.^†^ Eighty-six facilities participating in CDC’s Influenza-like Illness Surveillance Network^§^ and Oregon’s Electronic Surveillance System for the Early Notification of Community-based Epidemics^¶^ were randomized and approached sequentially with a goal of recruiting 18 facilities to provide 50 specimens each. Facilities were asked to submit random subsamples of deidentified sera from patients of all ages visiting any ambulatory, emergency, or inpatient health care setting and to include the specimen collection date and the patient’s date of birth. Specimens were stored according to instructions provided by the test manufacturer and transported to the Oregon State Public Health Laboratory for testing with the Abbott Architect Laboratories SARS-CoV-2 IgG immunoassay. Abbott Laboratories (Abbott Park, Illinois) reports a sensitivity of 96.8% at ≥14 days after a positive polymerase chain reaction test result and specificity of 99.1%–100% (*1*). Results from actual use support the reported analytical performance of this test (*2*).

Although 18 facilities were initially recruited, another facility was added through the same sequential approach because one facility was only able to submit 15 specimens. The facilities’ locations were approximately representative of the geographic distribution of Oregon’s population. During May 11–June 15, 2020, a total of 898 venous specimens (average from each facility = 47; range = 15–50) were collected from the 19 facilities; one specimen was discarded because of a laboratory error. This activity was reviewed by CDC and was conducted consistent with CDC policies and procedures, and institutional review board clearance was not required.** Stata (version 15.1; StataCorp) was used for all analyses.

Antibodies to SARS-CoV-2 were detected in nine of 897 specimens, yielding an unadjusted seroprevalence of 1.0% (95% confidence interval = 0.2%–1.8%). Antibodies were not detected in any specimens from the 29 persons aged ≤17 years. Seroprevalence generally increased with age (chi-squared test for trend, p = 0.049) (Table).

**TABLE:** Estimated seroprevalence of SARS-CoV-2 immunoglobulin G (IgG) antibodies among a convenience sample of deidentified serum specimens from 19 facilities participating in the Influenza-like Illness Surveillance Network, by age group* — Oregon, May 11–June 15, 2020

Age group (yrs)	No. of samples tested	SARS-CoV-2 IgG-positive^†^	
		No.	% (95% CI)
0–4	5	0	0 (0–52)
5–17	24	0	0 (1–14)
18–49	274	1	0.4 (0–2.0)
50–64	211	1	0.5 (0–2.6)
65–74	178	3	1.7 (0.3–4.8)
75–84	144	3	2.1 (0.4–6.0)
≥85	61	1	1.6 (0–8.8)
**Total**	**897**	**9**	**1.0 (0.2–1.8)**

**Abbreviation:** CI = confidence interval.

* Seroprevalence generally increased with age (chi-squared test for trend, p = 0.049).

^†^ Abbott Architect Laboratories SARS-CoV-2 IgG immunoassay. Abbott Laboratories (Abbott Park, IL) reports a sensitivity of 96.8% at ≥14 days after a positive polymerase chain reaction test result and specificity of 99.1%–100%.

The estimated seroprevalence of SARS-CoV-2 antibodies in a convenience sample of adult Oregonians was approximately 10 times the measured cumulative COVID-19 incidence obtained by nucleic acid testing, consistent with results from seven other U.S. states and geographic areas (*4*). This convenience sample, obtained from patients interacting with health care systems throughout the state, is not necessarily generalizable to the entire state population. Limitations of seroprevalence testing include false positivity in settings of low background prevalence such as Oregon, lack of antibody development by some infected persons, and in others, waning of antibodies to undetectable levels. The data suggest that a substantial number of COVID-19 cases in Oregon have gone undiagnosed and not reported and that a large portion of Oregon’s population remains susceptible to COVID-19 infection. Although the sample size was small, a pattern of increasing seroprevalence with age was observed. These findings are similar to those reported in a recent survey in neighboring Idaho (*1*). Follow-up surveillance studies are planned in Oregon to reassess cumulative incidence as the pandemic progresses.

## Footnotes

* https://www.pdx.edu/prc/population-reports-estimates.

† https://apps.who.int/iris/bitstream/handle/10665/331656/WHO-2019-nCoV-Seroepidemiology-2020.1-eng.pdf?sequence=1&isAllowed=y.

§ https://www.cdc.gov/flu/weekly/overview.htm#anchor_1539281266932.

¶ https://www.oregon.gov/oha/PH/DiseasesConditions/CommunicableDisease/PreparednessSurveillanceEpidemiology/essence/Pages/index.aspx.

** U.S. Department of Health and Human Services, Title 45 Code of Federal Regulations 46, Protection of Human Subjects.
